# Impact of pneumococcal vaccines use on invasive pneumococcal disease in Nunavik (Quebec) from 1997 to 2010

**DOI:** 10.3402/ijch.v73.22691

**Published:** 2014-01-17

**Authors:** Jean-Baptiste Le Meur, Brigitte Lefebvre, Jean-François Proulx, Serge Déry, Jacques Pépin, Philippe De Wals

**Affiliations:** 1 Quebec Heart and Lung Institute Research Centre, Quebec City, Canada; 2 Department of Social and Preventive Medicine, Laval University, Quebec City, Canada; 3 Quebec Public Health Laboratory (Laboratoire de santé publique du Québec), Quebec National Institute of Public Health (Institut national de santé publique du Québec), Ste-Anne-de-Bellevue, Canada; 4 Public Health Directorate, Nunavik Regional Board of Health and Social Services, Kuujjuaq, Canada; 5 Department of Microbiology and Infectious Diseases, University of Sherbrooke, Sherbrooke, Canada; 6 Etienne-Lebel Research Centre, Sherbrooke University Hospital, Sherbrooke, Canada

**Keywords:** immunization, epidemiology, *Streptococcus pneumonia*, paediatric vaccine, Inuit

## Abstract

**Background:**

In 2000, an outbreak of severe pneumonia caused by a virulent clone of serotype 1 *Streptococcus pneumoniae* was detected in the Nunavik region of Quebec. A mass immunization campaign was implemented in the spring of 2002, targeting persons ≥5 years of age and using the 23-valent pneumococcal polysaccharide vaccine (PPSV23). At the same time, the 7-valent pneumococcal conjugate vaccine (PCV7) was introduced into the routine immunization programme of infants, with catch-up for children up to 4 years of age.

**Objectives:**

To describe the epidemiology of invasive pneumococcal disease (IPD) in relation to PPSV23 and PCV7 use.

**Study design and methods:**

Retrospective analysis of IPD cases identified by the Quebec public health laboratory during the period 1997–2010.

**Results:**

A total of 82 IPD cases were identified during the study period. In adults, serotype 1 incidence decreased following the 2002 PPSV23 mass campaign but breakthrough cases continued to occur. Following PCV7 use in children, there was a decrease in the incidence of vaccine-type IPD and replacement by other serotypes in adults. In children, a marked decrease in the annual incidence of serotypes included in PCV7 was observed following PCV7 introduction: 162/100,000 in 1997–2001 vs. 10/100,000 in 2004–2010 (p<0.01). Concomitantly, the incidence of IPD caused by serotypes not included in PCV7 increased from 29/100,000 to 109/100,000 (p=0.11).

**Conclusion:**

The mass immunization campaign using the PPSV23 in 2002 and the introduction of PCV7 for the routine immunization of infants induced important modifications in the epidemiology of IPD. IPD rates in Nunavik remain much higher than in the southern part of the province both in children and adults. More effective pneumococcal vaccines are needed to eliminate geographic disparities in IPD risk.

Respiratory infections constitute a major public health problem in the northern communities of Canada and *Streptococcus pneumonia* (*Sp*) is the most important pathogen (1, 2). During the fall of 2000, an outbreak of severe pneumonia caused by a virulent clone of serotype 1 *Sp* started in the Inuit population of Nunavut and Nunavik and was identified in 2001 (3, 4). Nunavik is the most northerly region of the province of Quebec. It has approximately 11,000 inhabitants, 90% of whom are Inuit and who live in 14 communities scattered along the Hudson and Ungava bays. There is an outpatient healthcare centre in each community and 2 regional hospitals. Patients with a severe condition may be airlifted to tertiary care hospitals situated in the southern part of Quebec.

To control this outbreak, a mass immunization campaign was launched in the spring of 2002 (starting in February and completed by the end of June), targeting persons ≥5 years of age with the 23-valent pneumococcal polysaccharide vaccine (PPSV23, including serotypes 1, 2, 3, 4, 5, 6B, 7F, 8, 9N, 9V, 10A, 11A, 12F, 14, 15B, 17F, 18C, 19A, 19F, 20, 22F, 23F and 33F) (4). In April 2002, the 7-valent pneumococcal conjugate vaccine (PCV7, including serotypes 4, 6B, 9V, 14, 18C, 19F and 23F) was introduced for routine immunization of infants (4 doses) with catch-up for children up to the age of 4 years (5). The 10-valent pneumococcal conjugate vaccine (PCV10, including serotypes 4, 6B, 9V, 14, 18C, 19F, 23F, 1, 5, 7F) replaced PCV7 in the summer of 2009, and the 13-valent vaccine (PCV13, including serotypes 4, 6B, 9V, 14, 18C, 19F, 23F, 1, 5, 7F, 3, 6A, 19A) replaced PCV10 in January 2011 (6). The objective of this study was to describe the epidemiology of invasive pneumococcal disease (IPD) in relation to pneumococcal vaccines use in the population of Nunavik during the period 1997–2010.

## Material and methods

IPD is a reportable disease in Quebec. Specimens from normally sterile sites are cultured in hospital laboratories and *Sp* isolates are forwarded to the Quebec public health laboratory for characterization (7). A list of IPD cases identified during the 1997–2010 period was obtained. The immunization status of patients was extracted from records of the Nunavik Public Health Directorate. An inclusion criterion for IPD cases was that the patient was a resident of Nunavik. Population denominator figures were provided by the Quebec Statistics Institute (“Institut de la Statistique du Québec”). Incidence rates were computed as the number of IPD cases divided by the number of person-years (p-y) at risk. The study period was divided into 3 periods: pre-mass immunization campaign era (1997–2001), transition era (2002–2003) and post-outbreak and routine immunization era (2004–2010). Rates were compared using an exact two-sided test assuming a Poisson distribution with R software 2.12.1 (R Foundation for Statistical Computing, Vienna, Austria). As PCV7 provides cross-protection against serotype 6A, this serotype was included in PCV7-types (8).

The study was performed under the mandate of epidemiologic surveillance and programme evaluation of the Public Health Directorate of the Nunavik Region. As a consequence, the approval of a research ethics committee was not needed.

## Results

During the 14-year study period, a total of 82 IPD cases were reported in the Nunavik population representing 146,601 p-y of observation. All IPD cases were identified by culture: 79 from blood, 1 from cerebrospinal fluid and 2 from trans-thoracic aspirate. The overall IPD rate was 56/100,000 p-y. The age distribution of cases was as follows:<5 years=33 cases (40%); 5–19 years=18 cases (22%); 20–59 years=19 cases (23%); ≥60 years=12 cases (15%). About 56% (46/82) of cases were female. The age-specific IPD rates were: <5 years=169/100,000 p-y; 5–19 years=36/100,000 p-y; 20–59 years=27/100,000 p-y; ≥60 years=168/100,000 p-y.

**Fig. 1:**
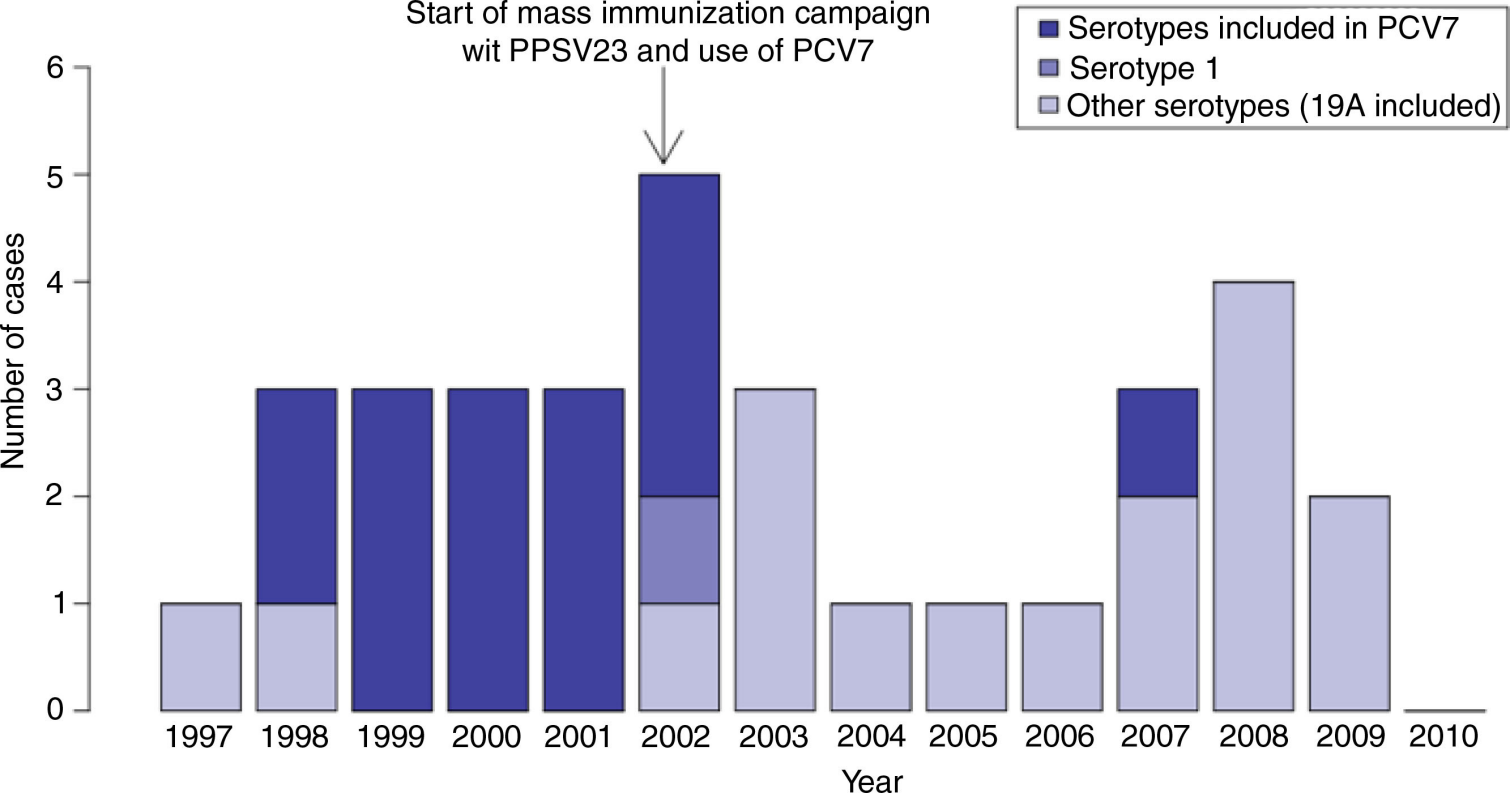
Number of cases of invasive pneumococcal disease in children less than 5 years of age in Nunavik: number of cases by year (total and by serotype), 1997–2010.

The distribution of IPD cases in children less than 5 years of age over the study period is shown in Fig. 1. Only one serotype 1 case was observed in this age group, in November 2002. Following the introduction of PCV7 in 2002, a decrease in the frequency of IPD was seen, and only one case per year was reported during the 2004–2006 period. Thereafter, the frequency of IPD cases rose again but declined following the introduction of PCV10 in the summer of 2009. During the years 2007–2009 (no cases in 2010), 4 serotype 19A (contained in PCV13 and PPSV23) cases were identified, out of a total of 9 cases.

**Fig. 2:**
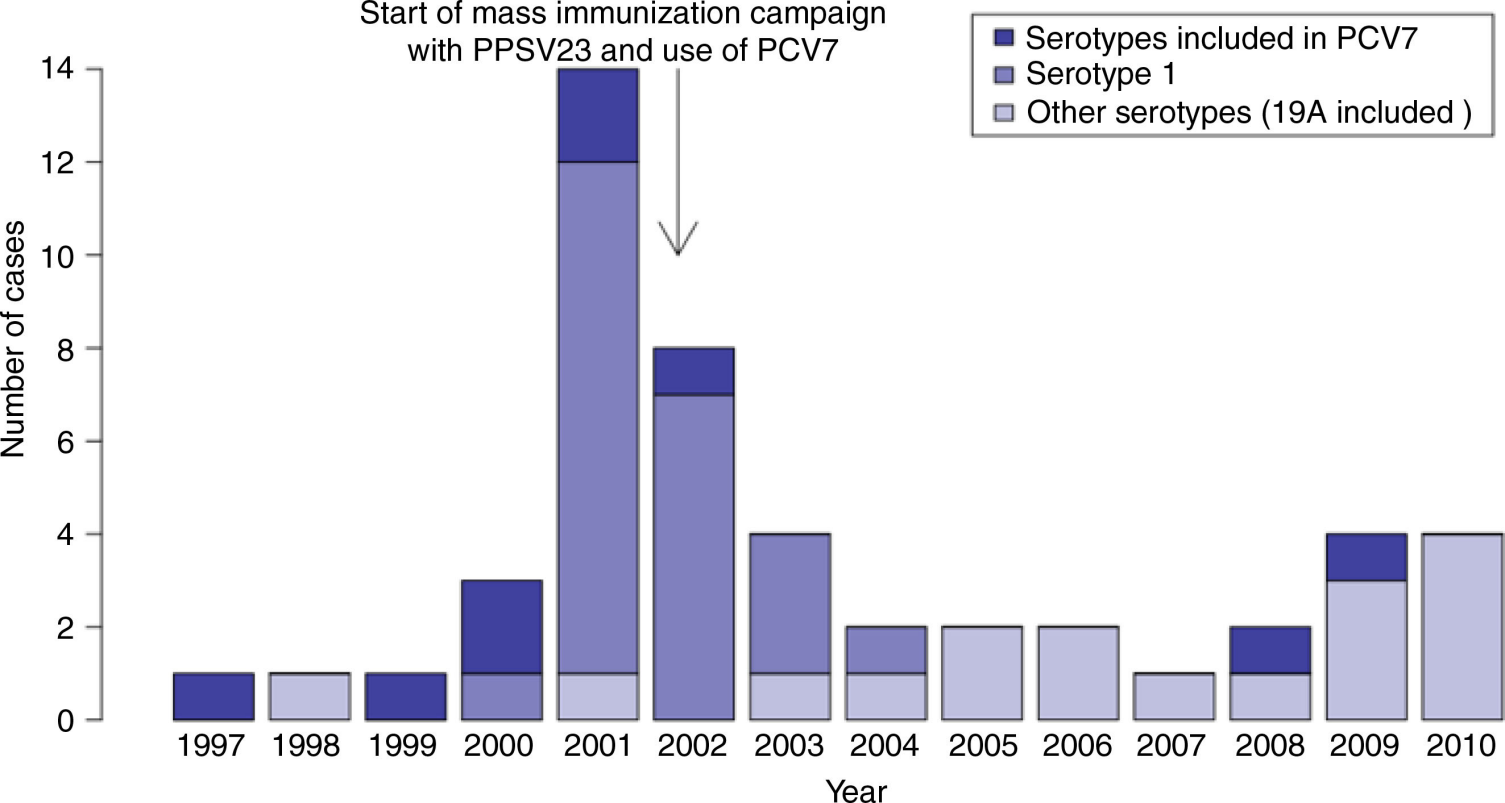
Number of cases of invasive pneumococcal disease in persons aged 5 years or more in Nunavik: number of cases by year (total and by serotype), 1997–2010.

The distribution of IPD cases in the population ≥5 years of age is displayed in Fig. 2. The outbreak caused by the virulent serotype 1 clone starting in 2000 is clearly seen. Following the mass immunization campaign implemented in February–June 2002, 9 serotype 1 cases were observed (5 in 2002, 3 in 2003 and 1 in 2004). Since the introduction of PCV7 in children in 2002, only 2 PCV7-type cases were observed in persons ≥5 years of age, including one serotype 4 case in 2009 in a teenager vaccinated with PPSV23 (containing serotype 4) in 2002.

IPD rates in children<5 years of age during the pre-PCV period (1997–2001), the PCV implementation period (2002–2003) and the post-implementation period (2004–2010) are shown in Table I. A marked and statistically significant decrease in the incidence of IPD caused by serotypes included in PCV7 was observed following PCV7 use: 162/100,000 p-y vs. 10/100,000 p-y (p<0.01). Concurrently, the incidence of IPD caused by serotypes not included in PCV7 increased, although this was not statistically significant: 29/100,000 p-y vs. 109/100,000 p-y (p=0.11). The observed rise during the 2002–2003 period (all serotypes included) is mainly due to cases that occurred prior to PCV7 introduction and large coverage.

**Table I:** Number of cases and incidence rate of invasive pneumococcal disease in children less than 5 years of age in Nunavik, Quebec, by period, 1997–2010

	Number of cases (% total)			Incidence rate per 100,000 person-years			
							Rate ratio 2004–2010 *vs*. 1997–2001 (p-value)
Serotypes	1997–2001	2002–2003	2004–2010	1997–2001	2002–2003	2004–2010	
All serotypes	13 (100%)	8 (100%)	12 (100%)	191	294	119	0.62 (0.32)
PCV7 types	11 (84%)	3 (38%)	1 (8%)	162	110	10	0.06 (<0.01)
Non-PCV7 types	2 (16%)	5 (62%)	11 (92%)	29	183	109	3.72 (0.11)
Serotype 1	0 (0%)	1 (12%)	0 (0%)	0	37	0	–
Serotype 19A	1 (8%)	2 (25%)	4 (33%)	15	73	40	2.71 (0.66)
Others	1 (8%)	2 (25%)	7 (59%)	15	73	70	4.74 (0.21)

In persons ≥5 years of age (Table II), the incidence of serotype 1 IPD was higher before than after the mass immunization campaign using the 23-valent polysaccharide vaccine: 29/100,000 p-y vs. 2/100,000 p-y (p<0.01). The incidence of infections caused by the 7 serotypes included in PCV7 also decreased following PCV use in children, although this was not statistically significant: 15/100,000 p-y vs. 3/100,000 p-y (p=0.07). The incidence of IPD caused by other serotypes (including serotype 19A) increased non-significantly: 5/100,000 p-y in 1997–2001 vs. 21/100,000 p-y in 2004–2010 (p=0.06). The increase was caused by a large variety of different serotypes (only 2 cases were serotype 19A in 2004–2010).

**Table II:** Number of cases and incidence rate of invasive pneumococcal disease in persons ≥5 years of age in Nunavik, Quebec, by period, 1997–2010

	Number of cases (% total)			Incidence rate per 100,000 persons-year			
							Rate ratio 2004–2010 *vs*. 1997–2001 (p-value)
Serotypes	1997–2001	2002–2003	2004–2010	1997–2001	2002–2003	2004–2010	
All serotypes	20 (100%)	12 (100%)	17 (100%)	49	68	25	0.51 (0.06)
PCV7 types	6 (30%)	1 (8%)	2 (12%)	15	5.5	3	0.20 (0.07)
PPSV23 types (not included in PCV7)	14 (70%)	10 (84%)	9 (53%)	34	57	13	0.39 (0.04)
Serotype 1	12 (60%)	10 (84%)	1 (6%)	29	57	1.5	0.05 (<0.01)
Serotype 19A	0 (0%)	0 (0%)	2 (12%)	0	0	3	+∞† (0.78)
Others	2 (10%)	0 (0%)	6 (35%)	5	0	8.5	1.8 (0.73)
Non-PCV7 or PPSV23 types	0 (0%)	1 (8%)	6 (35%)	0	5.5	9	+∞† (0.12)

† No case occurred during the period from 1997 to 2001, so by definition the rate ratio is infinite.

In the period following the mass vaccination campaign with PPSV23 (from July 2002 to December 2010), 69% (18/26) of IPD cases in persons ≥5 years of age were caused by serotypes included in PPSV23 (Table III). The immunization status of 14 out of the 18 PPSV23-type cases could be determined with certainty: 10 patients had been vaccinated with PPSV23 and the remaining 4 patients had not received this vaccine. The mean interval between vaccine administration and disease onset was 5.4 years (range 0.7–14.8 years). Interestingly, breakthrough cases caused by serotype 1 occurred shortly (0.7–2.4 years) following PPSV23 administration.

**Table III:** Number of cases of invasive pneumococcal disease in persons ≥5 years of age in Nunavik, Quebec (July 2002–December 2010), following the end of the mass immunization campaign using the 23-valent polysaccharide vaccine (PPSV23)

	PPSV23-types					
						Non-PPSV23-types	
Serotypes	1	7F	19A	Others	All		Total
Number of cases	9	2	2	5†	18	8††	26
Vaccinated with PPSV23	6	1	1	2	10	6	16
Mean interval in years between vaccination and disease onset (range)	1.5 (0.7–2.4)	13.8	8.6	11.2 (7.6–14.8)	5.4 (0.7–14.8)		

† Serotypes 3, 4, 9N, 10A and 20.

†† Serotypes 16F (2 cases), 6A, 15A, 15C, 21, 22A, 23A.

## Discussion

Laboratory surveillance of IPD in an Arctic region is particularly challenging. In Nunavik, for reasons of distances to laboratory and medical services (air transportation only and 9 communities out of 14 with no physician on site), antibiotics are often rapidly prescribed for patients with clinical signs of acute infections and blood cultures are not performed for all cases. Blood cultures can be performed only in the 2 regional hospitals, and by the time they get there many patients have already received antibiotics, decreasing the odds of a positive culture. It is thus likely that rates reported in our study are substantial underestimates of the real frequency of IPD in Nunavik. Nevertheless, the reported average IPD rate in the Nunavik region (56/100,000) is 5 times higher than the rate reported in the whole Quebec population (11/100,000) (6). Ethnic disparity in IPD rates has been observed between Caucasian and Alaska Native children (9); in our study, such a disparity could be due to several reasons: climate, household overcrowding and passive smoking. In Quebec, ethnicity is not recorded in medical charts and such analyses cannot be performed at the regional level. Moreover, the proportion of non-Inuit children in Nunavik is below 5%.

There is no computerized immunization registry in Nunavik and immunization records are kept in local health centres. It is thus difficult to assess precise vaccine coverage rates. Unpublished results of an on-going study show that 92% of children had received the recommended 4 PCV doses by 5 years of age. Another limitation is the small size of our study population. Results of statistical comparisons should thus be interpreted with care.

Following the PPSV23 mass campaign in the spring of 2002, the overall rate of serotype 1 IPD started to decrease in adults, 83.7% of whom had been vaccinated (5). However, vaccine failures were observed and the mass campaign did not prevent the emergence of new serotypes several years later, including those included in the polysaccharide vaccine. In a recent meta-analysis of randomized controlled trials, the estimate of the short-term efficacy of polysaccharide pneumococcal vaccines to prevent IPD cases caused by homologous serotypes in adults was 74% (10). Approximately the same level of vaccine efficacy was determined for Alaska Native adults by use of the indirect cohort method in an epidemiologic study performed in Alaska (11). However, protection wanes with time as demonstrated in a case–control study of US adults conducted from 1984 to 1990 (12). Furthermore, polysaccharide vaccines do not have a significant effect on *Sp* carriage and cannot induce herd protection (13). Better vaccines are needed for the prevention of pneumococcal infections in adults (14, 15).

PCV7 use in children was followed by a decrease in IPD rate, both in children and the general population. Although no vaccine failure was documented, the benefit of PCV7 was eroded by the emergence of non-vaccine serotypes. This phenomenon has also been observed in Alaska Native children (9, 16). The shift observed in the distribution of serotypes following PCV7 use is the result of selective bacterial replacement with non-vaccine serotypes (17). Serotype 19A was the most important emerging pathogen, which is consistent with other studies in Quebec and Alaska (6, 7, 16). However, serotype 7F (vaccine preventable in children less than 5 years old since 2009 and the use of PCV10) did not emerge among children in Nunavik, contrary to findings observed in southern Quebec (6).

In persons ≥5 years of age, the decrease in the incidence of infections caused by the 7 serotypes included in PCV7 is probably the result of herd protection induced by the vaccination of children. In Quebec, a small decrease of IPD incidence rate has been observed in all age groups after the implementation of a universal programme of immunization in children, with a marked decline in PCV7 types and an increase in non-vaccine types (6). In Nunavik, a large variety of non-vaccine serotypes have recently been observed among IPD cases, which complicates the selection of the optimal control strategy.

Following the introduction of PCV10 in the summer of 2009 for the routine immunization of infants, a further decrease in IPD incidence was observed in children. The same phenomenon was seen among children immunized with PCV10 in the whole Quebec population (18). PCV10 was only used during an 18-month period and therefore conclusions of increased effectiveness of the 10-valent as compared with the 7-valent vaccine should be made with caution. On-going surveillance will provide more information on the impact of PCV13.

## Conclusion

The mass immunization campaign in 2002 and the introduction of PCV7 for the routine immunization of infants induced important modifications in the epidemiology of IPD in the Nunavik region. Currently, IPD rates in Nunavik remain much higher than in the southern part of the province both in children and adults. More effective pneumococcal vaccines and socio-environmental strategies are needed to eliminate geographic disparities in IPD risk.

## References

[1] MacMillan HI, MacMillan AB, Offord DR, Dingle JL (1996). Aboriginal health. CMAJ.

[2] Degani N, Navarro C, Deeks SL, Lovgren M (2008). Invasive bacterial diseases in northern Canada. Emerg Infect Dis.

[3] Marcy JF, Roberts A, Lior L, Tam TWS, VanCaeseele P (2002). Outbreak of community-acquired pneumonia in Nunavut, October and November 2000. Can Commun Dis Rep.

[4] Proulx JF, Déry S, Jetté L, Ismaël J, Libman M, De Wals P (2002). Pneumonia epidemic caused by a virulent strain of *Streptococcus pneumoniae* serotype 1 in Nunavik, Quebec. Can Commun Dis Rep.

[5] Ndiaye AA, De Wals P, Proulx JF, Ouakki M, Jetté L, Déry S (2006). Impact of a mass immunization campaign to control an outbreak of severe respiratory infections in Nunavik, northern Canada. Int J Circumpolar Health.

[6] Douville-Fradet M, Amini R, Boulianne N, Khuc NH, De Wals P, Fortin E (2011). Impact du programme d'immunisation par le vaccin pneumococcique conjugué heptavalent (VPC-7) au Québec.

[7] Lefebvre B, Bourgault AM (2012). Programme de surveillance du pneumocoque: Rapport 2010.

[8] Whitney CG, Pilishvili T, Farley MM, Schaffner W, Craig AS, Lynfield R (2006). Effectiveness of seven-valent pneumococcal conjugate vaccine against invasive pneumococcal disease: a matched case-control study. Lancet.

[9] Wenger J, Zulz T, Bruden D, Singleton R, Bruce MG, Bulkow L (2010). Invasive pneumococcal disease in Alaskan children. Pediatr Infect Dis J.

[10] Moberley S, Holden J, Tatham DP, Andrews RM (2013). Vaccines for preventing pneumococcal infection in adults (Review). Cochrane Database Syst Rev.

[11] Singleton RJ, Butler JC, Bulkow LR, Hurlburt D, O'Brien KL, Doan W (2007). Invasive pneumococcal disease epidemiology and effectiveness of 23-valent pneumococcal polysaccharide vaccine in Alaska Native adults. Vaccine.

[12] Shapiro ED, Berg AT, Austrian R, Schroeder D, Parcells V, Margolis A (1991). The protective efficacy of polyvalent pneumococcal polysaccharide vaccine. N Engl J Med.

[13] Dagan R, Lipsitch M, Tuomanen EI, Mitchell TJ, Morrisson DA, Spratt BG (2004). Changing the ecology of pneumococci with antibiotics and vaccines. The pneumococcus.

[14] Benin AL, O'Brien KL, Watt JP, Reid R, Zell R, Katz S (2003). Effectiveness of the 23-valent polysaccharide vaccine against invasive pneumococcal disease in Navajo adults. J Infect Dis.

[15] Said MA, O'Brien KL, Pekka Nuorti J, Singleton R, Whitney CG, Hennessy TW (2011). The epidemiologic evidence underlying recommendations for use of pneumococcal polysaccharide vaccine among American Indian and Alaska Native populations. Vaccine.

[16] Bruce MG, Deeks SL, Zulz T, Bruden D, Navarro C, Lovgren M (2008). International circumpolar surveillance system for invasive pneumococcal disease, 1999–2005. Emerg Infect Dis.

[17] Hausdorff WP, Brueggemann AB, Hackell, Scott JAG, Siber GR, Klugman KP, Mäkelä PH (2008). Pneumococcal serotype epidemiology. Pneumococcal vaccines: the impact of conjugate vaccine.

[18] De Wals P, Lefebvre B, Markowski F, Deceuninck G, Defay F, Douville-Fradet M Impact of 2+1 pneumococcal conjugate vaccine program in the province of Quebec, Canada. Vaccine.

